# Nuclear heterogeneous nuclear ribonucleoprotein D is associated with poor prognosis and interactome analysis reveals its novel binding partners in oral cancer

**DOI:** 10.1186/s12967-015-0637-3

**Published:** 2015-08-30

**Authors:** Manish Kumar, Ajay Matta, Olena Masui, Gunjan Srivastava, Jatinder Kaur, Alok Thakar, Nootan Kumar Shukla, Ajoy RoyChoudhury, Meherchand Sharma, Paul G. Walfish, K. W. Michael Siu, Shyam Singh Chauhan, Ranju Ralhan

**Affiliations:** Department of Biochemistry, All India Institute of Medical Sciences, Room No. 3009, New Delhi, 110029 India; Alex and Simona Shnaider Laboratory of Molecular Oncology, Mount Sinai Hospital, 6-500, Toronto, ON M5G 1X5 Canada; Department of Chemistry, Centre for Research in Mass Spectrometry, York University, Toronto, ON Canada; Department of Otorhinolaryngology, All India Institute of Medical Sciences, New Delhi, India; Department of Surgery, Dr. B. R. A. Institute Rotary Cancer Hospital, All India Institute of Medical Sciences, New Delhi, India; Department of Dental Surgery, All India Institute of Medical Sciences, New Delhi, India; Department of Pathology, All India Institute of Medical Sciences, New Delhi, India; Department of Otolaryngology-Head and Neck Surgery, Joseph and Mildred Sonshine Family Centre for Head and Neck Diseases, Mount Sinai Hospital, Toronto, ON Canada; Department of Pathology and Laboratory Medicine, Mount Sinai Hospital, Joseph & Wolf Lebovic Health Complex, Toronto, ON M5G 1X5 Canada; Department of Otolaryngology-Head and Neck Surgery, University of Toronto, Toronto, ON Canada; Department of Medicine, Endocrine Division, Mount Sinai Hospital and University of Toronto, Toronto, ON Canada; Department of Chemistry, University of Windsor, Windsor, ON Canada

**Keywords:** hnRNPD/AUF1, Dysplasia, Oral cancer, Prognosis, mRNA stability, Interactome

## Abstract

**Background:**

Post-transcriptional regulation by heterogeneous ribonucleoproteins (hnRNPs) is an important regulatory paradigm in cancer development. Our proteomic analysis revealed hnRNPD overexpression in oral dysplasia as compared with normal mucosa; its role in oral carcinogenesis remains unknown. Here in we determined the hnRNPD associated protein networks and its clinical significance in oral squamous cell carcinoma (OSCC).

**Methods:**

Immunoprecipitation (IP) followed by tandem mass spectrometry was used to identify the binding partners of hnRNPD in oral cancer cell lines. Ingenuity pathway analysis (IPA) was carried out to unravel the protein interaction networks associated with hnRNPD and key interactions were confirmed by co-IP-western blotting. hnRNPD expression was analyzed in 183 OSCCs, 44 oral dysplasia and 106 normal tissues using immunohistochemistry (IHC) and correlated with clinico-pathological parameters and follow up data over a period of 91 months. Kaplan–Meier survival and Cox-multivariate-regression analyses were used to evaluate the prognostic significance of hnRNPD in OSCC.

**Results:**

We identified 345 binding partners of hnRNPD in oral cancer cells. IPA unraveled novel protein–protein interaction networks associated with hnRNPD and suggested its involvement in multiple cellular processes: DNA repair, replication, chromatin remodeling, cellular proliferation, RNA splicing and stability, thereby directing the fate of oral cancer cells. Protein–protein interactions of hnRNPD with 14-3-3ζ, hnRNPK and S100A9 were confirmed using co-IP-western blotting. IHC analysis showed significant overexpression of nuclear hnRNPD in oral dysplasia [p = 0.001, Odds ratio (OR) = 5.1, 95 % CI = 2.1–11.1) and OSCCs (p = 0.001, OR = 8.1, 95 % CI = 4.5–14.4) in comparison with normal mucosa. OSCC patients showing nuclear hnRNPD overexpression had significantly reduced recurrence free survival [p = 0.026, Hazard ratio = 1.95, 95 % CI = 1.0–3.5] by Kaplan–Meier survival and Cox-multivariate-regression analyses and has potential to define a high-risk subgroup among OSCC patients with nodal negative disease.

**Conclusions:**

Our findings suggest novel functions of hnRNPD in cellular proliferation and survival, besides RNA splicing and stability in oral cancer. Association of nuclear hnRNPD with poor prognosis in OSCC patients taken together with its associated protein networks in oral cancer warrant future studies designed to explore its potential as a plausible novel target for molecular therapeutics.

**Electronic supplementary material:**

The online version of this article (doi:10.1186/s12967-015-0637-3) contains supplementary material, which is available to authorized users.

## Background

Post-transcriptional regulation of mRNA stability and translation by RNA binding proteins (RBPs) is a key determinant of gene expression [1–3]. These RNA–protein interactions dictate the ultimate fate of the transcripts and are emerging as an important regulatory paradigm in cancer development [3, 4]. The mRNA decay kinetics is largely controlled by presence of specific *cis*-acting sequence and/or structural determinants within each transcript [5, 6]. About 16 % of all human protein coding genes are encoded by mRNAs that contain an adenylate-uridylate (AU)-rich element [ARE] motif within their 3′UTR [1, 2, 5, 6]. AU-rich RNA-binding factor (AUF1)/heterogeneous nuclear ribonucleoprotein D (hnRNPD) is an ARE-binding protein which regulates the mRNA stability of many genes involved in cell cycle, proliferation, survival, senescence and stress response [1, 2, 3, 4, 5, 7, 8, 9, 10, 11]. This protein harbors two RNA-binding domains arranged in tandem and a glycine-rich region in the C-terminus (2x RBD-Gly) and regulates the cellular half-life of many mRNAs by directly interacting with AREs in their 3′untranslated region [12–15]. Overexpression of hnRNPD in vivo resulted in deregulation of mRNAs including c-myc, c-jun, c-fos, and tumor necrosis factor-α (TNF-α) which promote tumorigenesis suggesting an oncogenic role of hnRNPD [1, 2, 3, 16, 17]. Increased hnRNPD expression also reduced the cell cycle checkpoint regulators p21 and p16^Ink4a^, a critical mediator of senescence [10, 18, 19]. Nuclear hnRNPD has been shown to activate the transcription promoter for telomerase catalytic subunit *Tert*, and links maintenance of telomere length and normal aging to attenuation of inflammatory cytokine expression and inhibition of cellular senescence [20].

Head and neck squamous cell carcinoma (HNSCC) ranks as the sixth leading cause of cancer related deaths worldwide [21]. HNSCCs often show heterogeneous pathologic and clinical features and diverse outcome [22, 23]. HNSCC is among the most morbid human malignancies and the quality of life in survivors is poor. Moreover, HNSCC patients often have recurrence of the tumor at the same site, or develop second primary tumors, frequently attributed to field cancerization [8]. Oral squamous cell carcinomas (OSCCs) comprise a large proportion of HNSCCs. The lack of clinically proven biomarkers limits therapeutic decisions to be solely based on tumor site and staging. However, tumors with similar clinical features can differ in disease outcome [24]. A better understanding of the molecular pathogenesis of OSCC is urgently needed for rigorous disease management. The development of OSCCs is often preceded by clinically distinct oral lesions such as leukoplakia or erythroplakia with histological evidence of squamous cell hyperplasia or dysplasia; on an average about one percent of these lesions transform to cancer annually. The oral lesions with histologically proven dysplasia are called Oral premalignant or potentially malignant lesions (OPLs). Identification of OPLs at high risk of progression to cancer is a high priority to enable early intervention, prior to development of frank malignancy for more effective disease management and improve the quality of life in survivors [25]. We reported overexpression of hnRNPD in human oral premalignant lesions by proteomic analysis [26]. In this study, interactome analysis was undertaken to gain an insight into hnRNPD associated protein–protein networks, by identifying its binding partners in oral cancer cells using immunoprecipitation followed by liquid chromatography—tandem mass spectrometry (LC–MS/MS). Bioinformatic analysis based cellular networks and pathways were identified and protein–protein interactions were confirmed using oral cancer cells. Further, we also determined the significance of hnRNPD overexpression in clinical specimens of oral dysplasia and cancer and correlated with disease outcome.

## Methods

### Cell culture

Oral squamous cell carcinoma (OSCC) cell line, SCC4 was obtained from American Type Culture Collection (ATCC), HSC2 (JCRB0622) from Health Science Research Resources Bank, Japan (HSRRB); Tu167 and MDA1986 were a kind gift from MD Anderson Cancer Centre (Houston, Texas). All cell lines were characterized using short tandem repeat polymorphism (STR) analysis. OSCC cells were grown in monolayer cultures in Dulbecco’s modified eagle medium (DMEM) (Sigma Aldrich, St. Louis, MO) supplemented with 10 % fetal bovine serum (FBS) (Sigma), 1 mM l-glutamine, 100 μg/ml streptomycin and 100 U/ml penicillin in a humidified incubator (5 % carbon-dioxide, 95 % air) at 37 °C as described previously [26, 27].

### Patients, tissue specimens, clinicopathological data and follow-up

The Institutional Human Ethics Committee of All India Institute of Medical Sciences (AIIMS), New Delhi, India, approved this study prior to its commencement (NO.IESC/T-261/03.06.2011). Tissue specimens were obtained from patients with oral dysplasia (n = 44) as revealed by H&E staining from Department of Otorhinolaryngology, All India Institute of Medical Sciences (AIIMS) and from 183 OSCC patients undergoing curative cancer surgery during the period 2002–2008, after obtaining patients’ consent, while 106 non-malignant oral tissues with histological evidence of normal epithelium constituted the normal group. Patient demographic, clinical, and pathological data were recorded in a pre-designed performa [27].

Of the 183 OSCCs, 144 cases could be followed-up in the head-and-neck cancer follow-up clinic at regular time intervals up to a maximum period of 91 months as of May, 2013. The patients were revisited clinically on a regular basis and the time to recurrence was recorded. Of these 144 OSCC patients, loco-regional relapse was observed in 60 cases (41.7 %), while 14 patients died (9.7 %) as determined from follow-up reports. If a patient died, the survival time was censored at the time of death; the medical history, clinical examination, and radiological evaluation were used to determine whether the death had resulted from recurrent cancer (relapsing patients) or from any other causes. Recurrence-free survivors (RFS) were defined as patients free from clinical and radiological evidence of local or regional recurrence or death at the time of the last follow-up.

### Real time-PCR analysis of hnRNPD mRNA levels in OSCCs and normal oral mucosa

For this study, hnRNPD mRNA level was determined in 12 paired tumor and normal tissue samples. Total RNA was extracted from a small portion of the biopsies OSCCs and normal oral tissues with Trizol reagent (Invitrogen, CA) according to the manufacturer’s protocol. The quality of the isolated RNA was tested by its optical density (260/280 ratio is 2.0). The expression of hnRNPD was quantified by real-time PCR (RT-qPCR). Total RNA (1 μg) was reverse-transcribed using Reverse transcriptase (Thermo Scientific, Waltham, MA, USA) using oligo-dT primers according to the manufacturer’s instructions. Real-time PCR reactions were performed and quantified by Maxima SYBR Green (Thermo Scientific, Waltham, MA, USA) using CFX96 Touch™ Real-Time PCR Detection System (BioRad, Hercules, CA, USA) using the ribosomal 18S gene as an internal control for normalisation. All assays were performed in triplicate in a 20 μl two-step reaction. The specificity of the amplified PCR products was assessed by melting curve analysis and agarose gel electrophoresis of a small aliquot of the reaction followed by staining with ethidium bromide. The efficiency of the qPCR reaction was measured in separate assays using cDNA obtained from total RNA of SCC4, HSC2, TU167 and MDA1986 oral cancer cell lines. The primer sequences are shown: hnRNPD–Sense: GCCTTTCTCCAGATACACCTGAAG; Antisense: CTTATTGGTCTTGTTGTCCA TGGG and 18S ribosomal–Sense: GTAACCCGTTGAACCCCATT, Antisense: CCA TCCAATCGGTAGTAGCG.

### Immunoblotting

Whole-cell lysates were prepared from OSCC cells (SCC4/HSC2/Tu167/MDA1986), oral normal tissues (n = 4), dysplasia (n = 2), and OSCC (n = 8) by homogenization in RIPA lysis buffer [26, 27]. Equal amounts of proteins (60 μg/lane) were resolved and electro-transferred onto polyvinylidene-difluoride (PVDF) membrane. After blocking blots were incubated with rabbit polyclonal hnRNPD antibody at 4 °C overnight. β-actin served as a control for equal protein loading in each lane. Membranes were incubated with their respective HRP-conjugated secondary antibody (DAKO Cytomation, Glostrup, Denmark), diluted at an appropriate dilution in 1 % BSA, for 2 h at room temperature. Protein bands were detected by the enhanced chemiluminescence method (ECL, Pierce, IL) on XO-MAT film.

### Immunoprecipitation

Oral cancer cells (SCC4/MDA1986) were lyzed in IP-lysis buffer as described [20]. Lysates were pre-cleared with Protein A-Sepharose (GE Healthcare Biosciences, Sweden), and immunecomplexes were obtained by incubation with polyclonal hnRNPD antibody and pulled down by incubating with Protein A-Sepharose. In negative controls, only Protein A Sepharose beads were added to eliminate proteins that bind non-specifically to the beads. Immunecomplexes were resolved on 10 % SDS-PAGE, stained with gel code blue and analyzed by reverse phase (RP)-liquid chromatography mass spectrometry (LC–MS/MS) as described [28, 29].

### Reverse phase (RP)-liquid chromatography mass spectrometry (LC–MS/MS)

The proteins bands were excised from gels and digested with trypsin as described [28, 29]. The digested peptides from each band were analyzed in duplicates using a Nanobore LC system (LC Packings, Amsterdam, Netherlands) and a QSTAR Pulsar mass spectrometer (Applied Biosystems/MDS SCIEX, Foster City, CA) in positive ion mode, externally calibrated with bovine serum albumin tryptic peptides [28, 29]. MS data were acquired in information-dependent acquisition (IDA) mode using Analyst QS 1.1 software (Applied Biosystems/MDS SCIEX). The LC–MS/MS was performed using a 1 s TOF–MS survey scan from 400 to 1500 Da, followed by four, 2 s product-ion scans, from 80 to 2000 Da, of the five most-abundant peaks. The collision energy (CE) was automatically controlled by the IDA CE parameter script. Switching criteria were set for ions with m/z ≥ 400 and <1500, charge states of +2 to +4, and abundances of ≥10 counts. Former target ions were excluded for 30 s, and ions within a 100-ppm window were ignored. To minimize redundancy in subsequent iterations, precursor ion exclusion (PIE) list was added to LC–MS/MS method as described earlier [28, 29].

### Identification of binding partners

LC–MS/MS data of each sample was used to identify proteins by searching a concatenated Swissprot/Panther database of 66082 distinct human protein entries (version June 2, 2010). The database was searched using Proteinpilot software, version 2.0.1 (AB SCIEX, Foster City), and the Paragon algorithm [30]. Protein identification was performed at a confidence threshold of 95 % (Proteinpilot Unused score ≥1.3) with methyl methanethiosulfonate (MMTS) selected as cysteine modification, and with the search option ‘emphasis on biological modifications’ checked. Peptide and protein summaries were generated. Only proteins identified with local false discovery rate (FDR) equal to, or less than, 5 % were considered for further analysis [28, 29]. Redundant proteins and peptides, proteins identified in reverse sequence and in negative controls (i.e. beads only) were removed from the list of identified proteins.

### Confocal laser scan microscopy (CLSM)

For CLSM, 5 × 10^4^ OSCC cells (SCC4/MDA1986) were plated on cover slips and grown for 24 h fixed in acetone: methanol mixture (1:1) at −20 °C for 20 min. [28]. Cells were permeabilized with PBS-0.1 % Tween 20, non-specific binding blocked with 5 % BSA for 1 h; cells were incubated with rabbit polyclonal hnRNPD (ab50692)/mouse monoclonal hnRNPK (ab23644, Abcam, CA) antibody at 4 °C overnight. Expression of proteins was determined using fluorescein isothiocyanate (FITC)/TRITC-labeled secondary antibodies (DAKO Cytomation, Denmark) [27].

### Immunohistochemistry of hnRNPD, hnRNPK and 14-3-3ζ in oral tissues and scoring

Paraffin-embedded tissue sections were deparaffinized, antigen was retrieved, endogenous peroxidase activity was quenched with hydrogen peroxide (0.3 % v/v) and non-specific binding blocked with 1 % bovine serum albumin (BSA). The slides were incubated with either rabbit polyclonal anti-hnRNPD antibody (1 μg/ml, ab50692, Abcam, CA) or mouse monoclonal anti-hnRNPK antibody (ab23644) or rabbit polyclonal 14-3-3ζ antibody (sc-1019) for 16 h at 4 °C. The primary antibody was detected using the Dako Envision kit (Dako CYTOMATION, Glostrup, Denmark) with diaminobenzidine as the chromogen and counterstained with hematoxylin [26, 27]. The sections were evaluated by light microscopy and scored using a semi-quantitative scoring system for both staining intensity (nuclear/cytoplasmic) and percentage positivity as described earlier [26, 27]. The tissue sections were scored based on the % of immunostained cells as: 0–10 % = 0; >10–30 % = 1; >30–50 % = 2; >50–70 % = 3 and >70–100 % = 4. Sections were also scored semi-quantitatively on the basis of staining intensity as negative = 0; mild = 1; moderate = 2; intense = 3. Finally, a total score was obtained by adding the score of percentage positivity and intensity giving a score range from 0 to 7. IHC score thus obtained for different proteins were subjected to statistical analysis.

### Statistical analysis

The immunohistochemical data were subjected to statistical analyses using the SPSS 20.0 software (Chicago, IL, USA). Sensitivity and specificity was calculated using receiver operating characteristic (ROC) analyses. Based on sensitivity and specificity values a cut-off ≥4 was defined as positive criterion for hnRNPD (nuclear/cytoplasmic). The relationships between hnRNPD and clinicopathological parameters were tested using Chi Square and Fischer’s exact test. Two-sided p-values were calculated and p < 0.05 was considered to be significant. Similarly, positive predictive value (PPV) and negative predictive value (NPV) were calculated for hnRNPD overexpression in oral lesions and OSCCs in comparison with normal oral mucosa. The correlation of hnRNPD staining with patient survival was evaluated using life tables constructed from survival data with Kaplan–Meier plots and Cox regression multivariate analysis. In order to confirm the association among hnRNPD, hnRNPK and 14-3-3ζ overexpression in clinical specimens of OSCCs, we performed Kappa analysis to determine the agreement of association between these proteins using their IHC scores. One of the most important features of the Kappa statistical analysis is its ability to measure the degree of agreement or reliability of agreement [26–28].

## Results

### Expression of hnRNPD in oral cancer cells and tissues

**Fig. 1:**
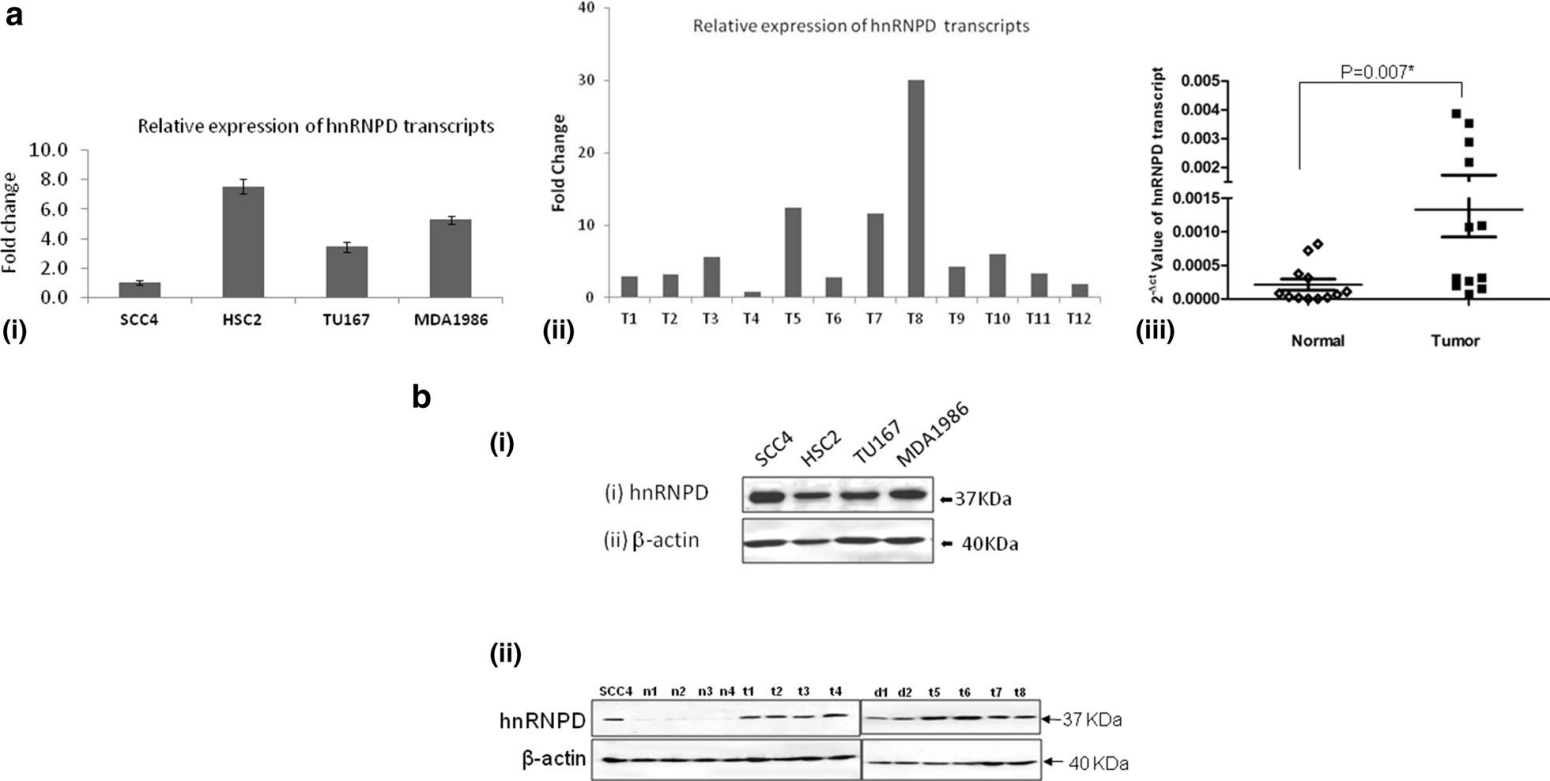
Analysis of hnRNPD expression in oral tissues. **a** Quantitative real time PCR (qPCR) analysis of hnRNPD transcripts in oral cancer cells, OSCCs and normal tissues. *i* Analysis of hnRNPD mRNA levels in oral cancer cell line. Total cellular RNA from various oral cancer cell lines (SCC-4, HSC-2, Tu-167, MDA-1986) were isolated, reverse transcribed and subjected to real time PCR analysis using specific hnRNPD primers. Simultaneously real time PCR for 18S ribosomal RNA was performed and served as an internal control for normalization. After real time PCR the 2^−Δct^ value of hnRNPD mRNA obtained in SCC-4 cells was assigned a value of 1 and the fold increase in other cell lines was calculated relative to this. Histogram shows the relative abundance of hnRNPD transcripts in SCC4, HSC2, Tu167 and MDA1986. *ii* Expression of hnRNPD transcripts in 12 random OSCCs samples and their paired normal tissues. Total cellular RNA was isolated from OSCCs and their paired normal tissues, reverse transcribed, and subjected to real time PCR analysis using specific hnRNPD primers. Parallel real time PCR for 18S ribosomal RNA was performed which served as an internal control for normalization of hnRNPD mRNA levels in OSCC and paired normal tissues. After normalization the fold increase in the levels of hnRNPD transcripts in each OSCC sample over their respective paired normal tissue was calculated and plotted individually. hnRNPD mRNA levels were significantly higher in oral OSCCs patients as compared to their paired normal oral tissue samples (p = 0.007, Mann–Whitney U test). *Bar* graph data is represented by fold changes of OSCCs samples after normalization with paired normal tissue samples. *iii* A representation of scatter plot after normalized 2^−Δct^ values of each OSCCs and paired normal tissue samples. **b** Western blot analysis. Photomicrograph showing protein expression of hnRNPD in (*i*) oral cancer cell lines (SCC4, HSC2, Tu167 and MDA1986); *ii* tissue lysates obtained from normal oral mucosa (n1–n4), oral dysplasia (d1, d2) and OSCCs (t1–t8).* Panel* shows increased expression of hnRNPD in dysplasia (d1, d2) and OSCCs (t1–t8) as compared to normal mucosa (n1–n4). Whole cell lysates prepared from SCC4 cells was used as a positive control. β-actin was used as loading control (*lower panel*)

Real time PCR and Western blotting were performed to determine the expression of hnRNPD transcripts and protein expression levels respectively in oral cancer cells (SCC4, HSC2, Tu167 and MDA1986), OSCCs and normal oral mucosa tissues. Real time PCR analysis revealed increased hnRNPD transcript levels in oral cancer cell lines (SCC4, HSC2, Tu167 and MDA1986) and OSCC tissue samples in comparison with normal oral epithelium (Fig. 1a, p = 0.007). These data were further verified using Western blot analysis.

Western blot analysis showed a single intense band of 37 kDa in all the oral cancer cell lines tested (SCC4, HSC2, Tu167 and MDA167, Fig. 1b), dysplasia (d1, d2) and OSCCs (t1–t8) demonstrating the presence of only p37/AUF1 isoform of hnRNPD. Faint or no expression of hnRNPD was observed in normal oral tissues (n1–n4), while an intense band was observed in OSCCs, thus confirming hnRNPD protein overexpression in OSCCs (Fig. 1b).

### Identification of binding partners of hnRNPD in OSCC cells

To gain an insight into the role of hnRNPD in OSCCs, we identified its binding partners in oral cancer cell lines (SCC4 and MDA1986). Immunoprecipitates obtained from SCC4 and MDA1986 cells using hnRNPD specific antibody were separated on 10 % SDS-PAGE, stained with gel-code blue, 35 protein bands were excised from the immunoprecipated sample and from the mock treated sample, digested with trypsin and subjected to RP-LC–MS/MS analysis for identification of proteins (Additional file 1: Figure S1A, Additional file 2: Figure S1B). Our novel approach using multiple iterations and development of precursor ion exclusion (PIE) list for protein identification revealed 345 binding partners of hnRNPD in oral cancer cell lines (Additional file 3: Table S1). Our approach revealed interactions of hnRNPD with 17 members of hnRNP family including hnRNPA2/B1, hnRNPK, hnRNPU, hnRNPG suggesting that hnRNPD forms heterodimers with its family members (Additional file 3: Table S1). hnRNPD also showed interactions with proteins involved in short-interfering-RNA (RNAi)-mediated gene silencing (EIF2C1, EIF2C2, EIF2C3), DNA repair (XRCC5, XRCC6), chromatin remodeling (SMARCC1, SMARCC2, SMARCB1; histone family of proteins including H1, H2A, H2B and H4), tumor protein 63 (TP63), transcription factors (zinc finger domain proteins, ZC3HAV1, ZCCHC8), cell signaling proteins (IGFBP, G3BP1, GNB2L1, NCL), nuclear-shuttling proteins (14-3-3ζ), S100A9 (calcium binding protein) and several other proteins involved in RNA splicing, stability and decay (ribosomal proteins 28S, 40S and 60S; ATP dependent RNA helicases, mRNA cap guanine-N7 methyltransferase, RNMT, RAE1) supporting its function in translation (Additional file 3: Table S1).

### Network analysis of hnRNPD protein interactions

**Fig. 2:**
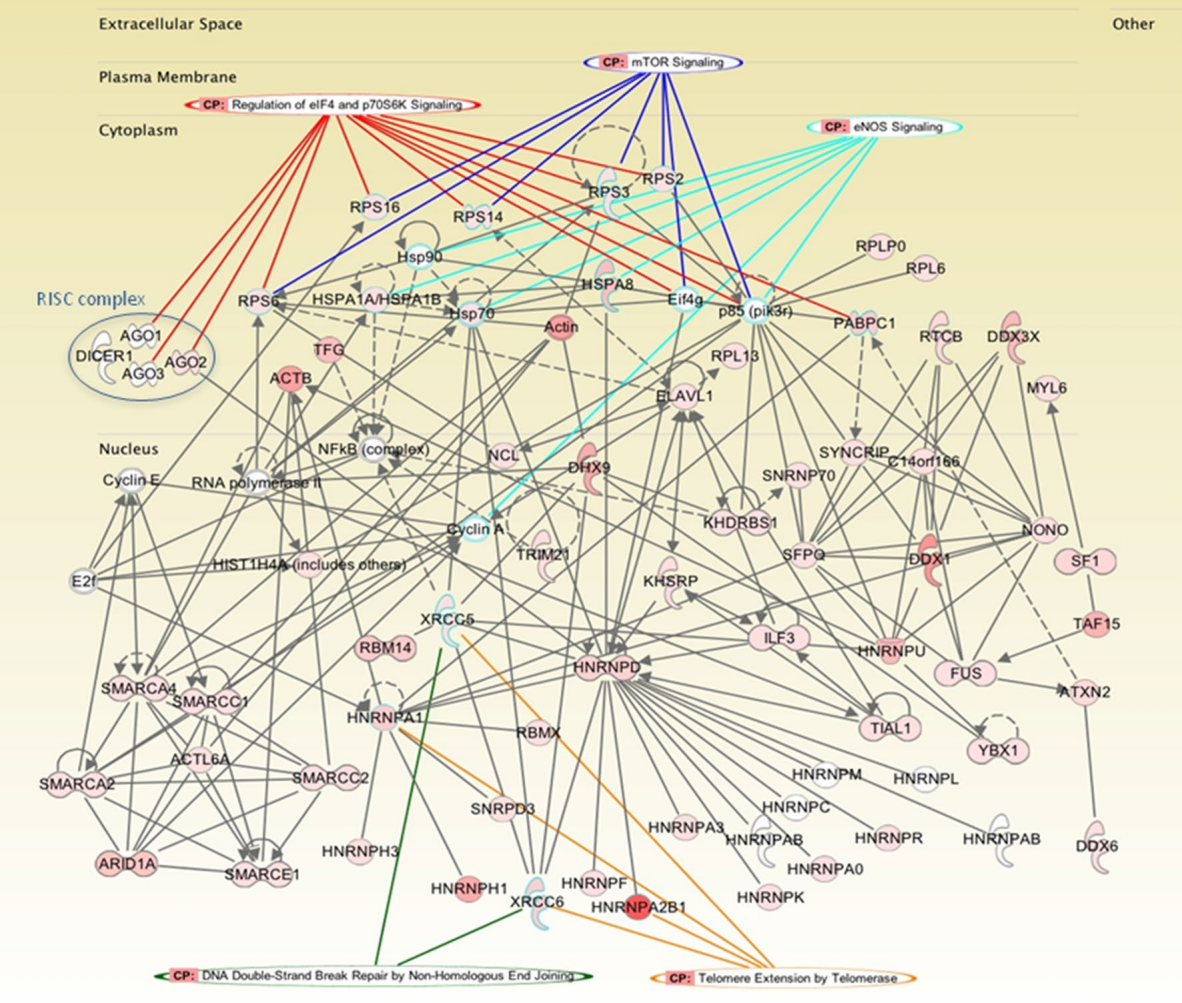
IPA analysis based hnRNPD networks in oral cancer. Network analysis obtained using Ingenuity Pathway Analysis (IPA) demonstrating interactions (direct/indirect) of hnRNPD with proteins involved in cellular signaling pathways including mTOR and eNOS; Double strand DNA repair mechanism (XRCC5, XRCC6); Chromatin remodeling (SMARCC1, SMARCC2, SMARCB1, SMARCD1, SMARCD2, SMARCE1 and histone family of proteins including H1, H2A, H2B and H4); Telomere maintenance (hnRNPA2/B1, XRCC5, XRCC6, hnRNPA1); RISC (short-interfering-RNA-mediated gene silencing) (EIF2C1, EIF2C2, EIF2C3), RNA splicing, stability, translation and decay (ribosomal proteins 28S, 40S and 60S; ATP dependent RNA helicases, mRNA cap guanine-N7 methyltransferase, RNMT, RAE1, PTBP1, FIP1L1, DHX15, RSL1D1, RBMS1, RBM12B)

Ingenuity pathway analysis (IPA) was carried out to generate the network of proteins identified in the hnRNPD interactome analysis. The criteria applied for the search of major biological function categories were maximum number of proteins and a significant *p* value. Our network analysis revealed novel signaling proteins that may interact (directly/indirectly) with hnRNPD and/or regulate its associated networks (Fig. 2) in addition to the proteins identified in our proteomics analysis. IPA analysis revealed 90 canonical signaling pathways significantly associated with hnRNPD (p-value <0.001, Additional file 3: Table S2). The binding partners of hnRNPD identified herein were also classified on the basis of their cellular functions (Additional file 3: Figure S1B). Cleavage and polyadenylation of pre-mRNA, nucleotide excision repair pathway, EIF2 signaling, mammalian target of rapamycin (mTOR) signaling, regulation of eukaryotic initiation factor 4 (eIF4) and p70S6K signaling, nitric oxide synthase (NOS), and estrogen receptor signaling emerged as significant pathways associated with hnRNPD (Fig. 2; Additional file 3: Table S2).

### Verification of interactions between hnRNPD and 14-3-3ζ, hnRNPK and S100A9

**Fig. 3:**
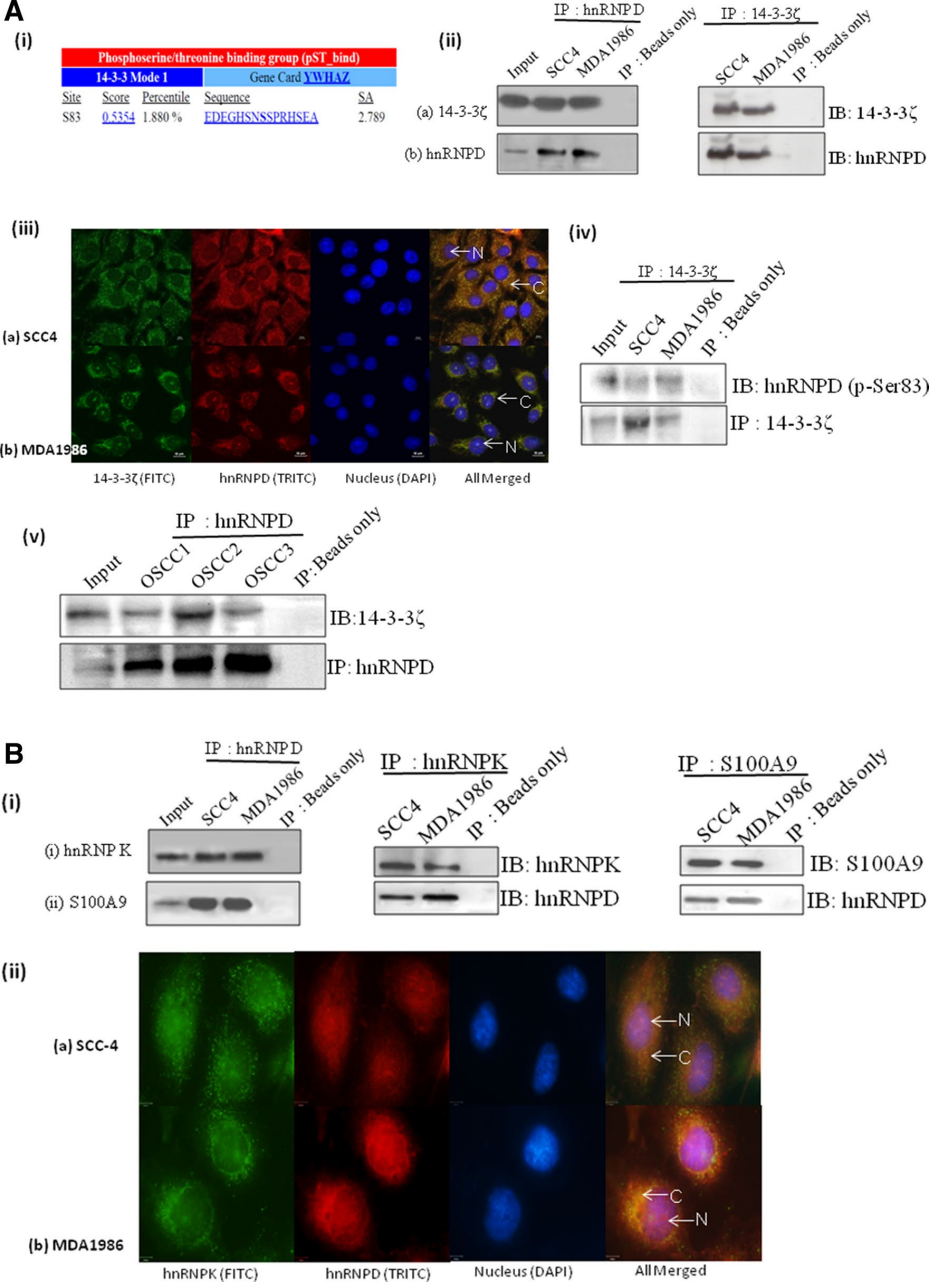
Verification of hnRNPD binding partners in oral cancer. **A**, *i* 14-3-3 binding motif of hnRNPD. Photomicrograph showing presence of 14-3-3 binding motif, Mode 1 in hnRNPD polypeptide sequence as revealed by http://www.motif.scan.mit.edu; *ii* Western blot showing presence of 14-3-3ζ and hnRNPD proteins in immunoprecipitates of hnRNPD/14-3-3ζ obtained from OSCC cells (SCC4 and MDA1986) while no band was seen in negative controls (i.e. beads only control). Input represents whole cell lysates from MDA1986 used as positive control for Western blotting; *iii* Confocal laser scan microscopy (CLSM) demonstrating nuclear and cytoplasmic expression of 14-3-3ζ (*green*, FITC) and hnRNPD (*red*, TRITC) in oral cancer cells *a* SCC4 and *b* MDA1986 cells. *Blue* colored DAPI was used as nuclear stain. Merged photomicrographs represent the co-localization of hnRNPD with 14-3-3ζ in oral cancer cells; *iv* Confirmation of binding of 14-3-3ζ at p-Ser83 of hnRNPD in oral cancer cells. Western blot revealed the presence of p-Ser83 of hnRNPD in immunoprecipitates obtained by using anti-14-3-3ζ antibody from SCC4 and MDA1986 cell line; **B** Western blot showing presence of hnRNPK and S100A9 proteins in immunoprecipitates of hnRNPD obtained from OSCC cells (SCC4 and MDA1986) while no band was seen in negative controls. Input represents whole cell lysates from MDA1986 used as positive controls; *ii* Confocal laser scan microscopy (CLSM) demonstrating nuclear and cytoplasmic expression of hnRNPK (*green*, FITC) and hnRNPD (*red*, TRITC) in oral cancer cells *a* SCC4 and *b* MDA1986 cells. *Blue* colored DAPI was used as nuclear stain. Merged photomicrographs represent the co-localization of hnRNPD with hnRNPK in oral cancer cells

As a first step for verification of interactions between hnRNPD and 14-3-3ζ observed in proteomics analysis, we performed motif scan (14-3-3—Mode 1/Mode 2) using software available online at http://www.motifscan.mit.edu as described earlier [26]. Motif search showed the presence of 14-3-3 binding motif Mode1 (EDEGH**S**NSSPRHSEA) in hnRNPD protein (Fig. 3A, i). Co-immunoprecipitation assays followed by western blot analysis were performed for both hnRNPD and 14-3-3ζ protein to verify their direct interactions in oral cancer cells (SCC4/MDA1986). As shown in Fig. 3A, 14-3-3ζ was detected in immunocomplexes of hnRNPD obtained from SCC4 and MDA1986 cells. Similarly, using reverse-IP experiments, hnRNPD was detected in immunocomplexes of 14-3-3ζ obtained from both the oral cancer cell lines (SCC4/MDA1986), while no band was detected in negative controls (Fig. 3A, ii). This interaction was further evident by co-localization of both these proteins in oral cancer cells (Fig. 3A, iii). Further, co-IP assays followed by western blotting using phospho-specific hnRNPD (^83^Ser) antibody showed the presence of 14-3-3ζ in both the oral cancer cell lines (SCC4 and MDA1986, Fig. 3A, iv). In addition, our results also demonstrated presence of 14-3-3ζ protein in hnRNPD immunocomplexes, obtained from OSCC tissue lysates (Fig. 3A, v).

Using similar approach, we verified the interaction of hnRNPD with hnRNPK and S100A9 in oral cancer cells. Western blotting showed presence of hnRNPK and S100A9 in hnRNPD immunoprecipitates and these findings were also confirmed using reverse—IP (Fig. 3B, i). Confocal laser scan microscopy (CLSM) analysis confirmed cytoplasmic and nuclear co-localization of hnRNPD with hnRNPK in OSCC cell lines, SCC4 and MDA1986 (Fig. 3B, ii).

### Immunohistochemical analysis of hnRNPD expression in oral normal mucosa, dysplasia and OSCCs

**Fig. 4:**
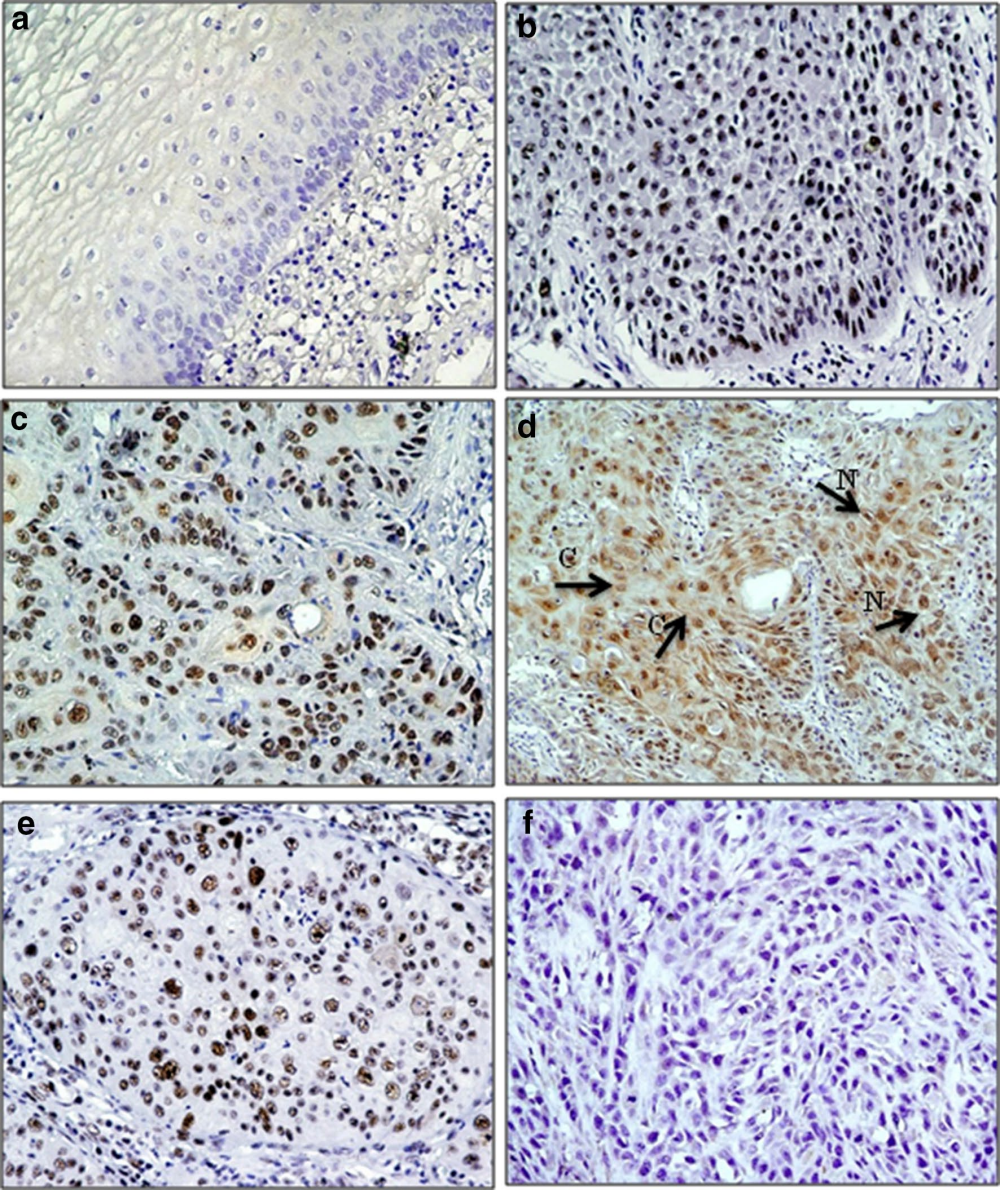
Immunohistochemical analysis of hnRNPD in oral tissues. Paraffin-embedded sections of histologically normal mucosa, oral dysplasia and OSCC were stained using anti-hnRNPD polyclonal antibody as described in “Methods” section.* Panel* represents **a** normal oral mucosa showing no hnRNPD immunostaining; **b** Dysplasia section illustrating nuclear hnRNPD staining in epithelial cells; **c** OSCC section illustrating nuclear hnRNPD staining in tumor cells; **d** OSCC section illustrating nuclear (*N*) and cytoplasmic (*C*) hnRNPD staining in tumor cells; **e** Lung cancer tissue section showing nuclear hnRNPD immunostaining; **f** OSCC section used as a negative control, showing no hnRNPD immunostaining in tumor cells; (**a**–**e** original magnification ×200)

Further, we performed immunohistochemistry to evaluate the clinical relevance of hnRNPD overexpression in tissue specimens of oral dysplasia and OSCCs. We performed immunohistochemistry of hnRNPD protein in oral normal mucosa (n = 106), dysplasia (n = 44) and OSCC (n = 183) tissues and analyzed its expression separately in both nucleus and cytoplasm of epithelial cells in each tissue section. The immunoreactivity score of 4 was selected as the threshold of hnRNPD immune-positivity based on the high sensitivity and specificity (>75 %) obtained with this cut off score in ROC analysis. Notably, weak or no detectable nuclear hnRNPD immunostaining was observed in 86/106 cases (81.1 %) normal tissues (Fig. 4a), while 20 cases only (18.9 %) showed moderate nuclear expression in epithelial cells of basal/suprabasal layer. Increased nuclear expression of hnRNPD was observed in 54.5 % dysplasia (24 of 44 cases, p < 0.001). Among different grades of dysplasia, 62.5 % of mild, 33.3 % moderate and 33.3 % severe dysplasia showed overexpression of nuclear hnRNPD (Table 1, Fig. 4c). Of 183 OSCCs analyzed in this study, 65.6 % cases showed increased nuclear hnRNPD expression in tumor cells as compared to normal oral mucosa (p < 0.001, odds ratio (OR) = 8.1, 95 % CI = 4.6–14.5; Table 1 and Fig. 4d). Overexpression of nuclear hnRNPD showed significant correlation with increasing tumor size (p = 0.005, OR = 2.4, 95 % C.I. = 1.3–4.6) and tumor stage (p = 0.02, OR = 2.3, 95 % C.I. = 1.0–5.2, Table 1). Thirteen of 183 (7.1 %) OSCCs showed cytoplasmic immunostaining of hnRNPD (Fig. 4d), but no significant association of cytoplasmic hnRNPD was observed (data not shown). Lung cancer tissue sections used as positive controls showed strong nuclear hnRNPD expression (Fig. 4e), while no immunostaining was observed in tissue sections used as negative controls where the primary antibody was replaced by isotype specific IgG (Fig. 4f). No significant alterations were observed in cytoplasmic hnRNPD expression in these tissues (data not shown).

**Table 1:** Analysis of hnRNPD protein expression and correlation with clinicopathological parameters

Clinicopathological features	Total cases N	Nuclear n	Positivity (%)	p value	Odd’s ratio	(95 % CI)
Normal	106	20	(18.9)			
Dysplasia	44	24	(54.5)	*<0.001* ^a^	*5.4*	*(2.5–11.7)*
Mild	31	18	(58.0)			
Moderate	10	4	(40)			
Severe	3	1	(33.3)			
OSCCs	183	120	(65.6)	*<0.001* ^b^	*8.1*	*(4.6–14.5)*
Age (median 45 years) (range 15–85 years)						
<45	64	47	(73.4)			
≥45	119	73	(61.3)	0.10	0.5	(0.2–1.1)
Gender						
Male	149	102	(68.5)			
Female	34	18	(52.9)	0.08	1.9	(0.9–4.1)
Differentiation						
WDSCC	89	57	(64.0)			
MDSCC	86	58	(67.4)			
PDSCC	8	5	(62.5)	0.87		–
Tumor size						
T_1_ + T_2_	65	34	(52.3)			
T_3_ + T_4_	118	86	(72.9)	*0.005*	*2.4*	*(1.3–4.6)*
Tumor stage						
I + II	31	15	(48.4)			
III + IV	152	105	(69.1)	*0.02*	*2.3*	*(1.0–5.2)*
Nodal status						
N_0_	61	37	(60.7)			
N_1–4_	122	83	(68.0)	0.32	1.3	(0.7–2.6)
Habits*						
Non consumer	33	27	(69.7)			
Tobacco consumer	150	97	(64.7)	0.58	0.7	(0.3–1.7)

* Tobacco consumption habits include tobacco chewing and/or smoking of bidi or cigarettes, chewing of betel quid, areca nut or pan masala

^a^Normal vs. dysplasia

^b^Normal vs. OSCCs

### Evaluation of nuclear hnRNPD overexpression as a prognostic marker for OSCC

**Fig. 5:**
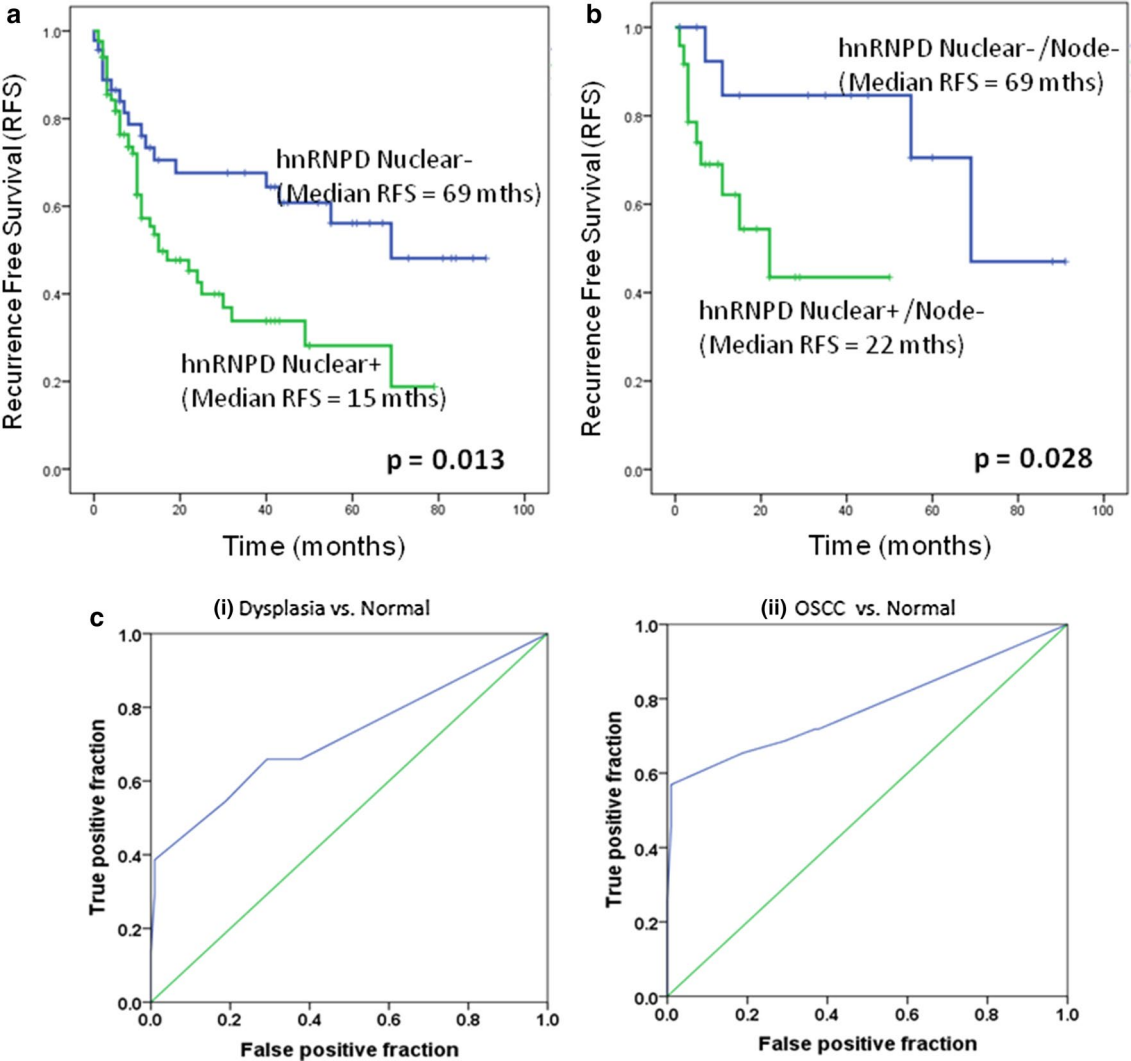
Evaluation of hnRNPD overexpression as a prognostic marker for OSCC. **a** Evaluation of nuclear hnRNPD as a prognostic marker. Based on sensitivity and specificity values a cut-off ≥4 was defined as positive criterion for hnRNPD (nuclear). Kaplan–Meier estimation of recurrence-free survival (RFS; no recurrence/metastasis) in OSCC patients showing nuclear immunostaining of hnRNPD was 15 months as compared to 69 months for the patients showing low scores for nuclear hnRNPD immunostaining (p = 0.013). **b** Kaplan–Meier estimation of recurrence-free survival, in node negative OSCC patients showing nuclear immunostaining of hnRNPD was median RFS = 22 months as compared to 69 months for the patients showing low scores for nuclear hnRNPD immunostaining and absence of node (p = 0.028). **c** Evaluation of nuclear hnRNPD as a diagnostic marker for distinguishing oral dysplasia and OSCCs from normal oral tissues.* Panel* shows ROC analysis for *i* Oral dysplasia vs. normal and *ii* OSCCs vs. normal oral tissues

We determined association of nuclear hnRNPD with survival of OSCCs using Kaplan–Meier (KM) analysis followed by multivariate Cox regression analysis. Kaplan–Meier survival analysis showed significantly reduced recurrence-free survival (median RFS = 15 months, p = 0.013) in OSCC patients harboring increased nuclear hnRNPD expression as compared to median RFS of 69 months in patients showing no or low nuclear hnRNPD (Fig. 5a). Notably, among OSCC patients with negative nodal status (i.e. N_0_) hnRNPD nuclear expression emerged as a significant predictor for recurrence with median RFS = 22 months as compared to patients who were negative for histopathological involvement of nodes and showed low score on nuclear hnRNPD (median RFS = 69.0 months, p = 0.028, Fig. 5b). However, nuclear hnRNPD did not show any significant association with recurrence among OSCC patients with positive nodes at time of surgery (p = 0.141).

Cox regression multivariate analysis was carried out to determine the prognostic potential of nuclear hnRNPD for OSCCs in comparison with the other clinical parameters including age, gender, histological grade, tumor size, nodal status and stage (Table 2). Nuclear hnRNPD emerged as the most significant marker for predicting recurrence in OSCC patients [p = 0.026, Hazard ratio (HR) = 1.95, 95 % CI = 1.0–3.5].

**Table 2:** Evaluation of nuclear hnRNPD as a prognostic marker of OSCCs

S. no.	Clinicopathological parameter	p value
1	Histopathologic grade	0.233
2	Tumor size	0.504
3	Tumor stage	0.340
4	Nodal status	0.359
5	hnRNPD (nuclear)	*0.026* ^a^
6	Tobacco habits	0.709
7	Gender	0.439
8	Age	0.050

Italicized value correspond to hnRNPD which immerged as an independent prognostic marker OSCC

^a^Cox multivariate analysis: Hazard ratio (HR) 1.95, 95 % CI 1.0–3.5

Further, ROC analysis showed an area under the curve (AUC) of 0.725 and 0.782 for dysplasia and OSCCs respectively for nuclear hnRNPD overexpression with high specificity, implicating its potential utility in distinguishing these tissues from normal epithelium (Table 3, Fig. 5c). Sensitivity, positive predictive value (PPV) and negative predictive value (NPV) for distinguishing dysplasia and OSCCs from normal oral mucosa are given in Table 3.

**Table 3:** Evaluation of hnRNPD as a diagnostic marker

hnRNPD	Specificity	Sensitivity	PPV	NPV	AUC
Dysplasia vs. normal	81.1	54.5	81.0	56.0	0.723
OSCCs vs. normal	81.1	65.5	86.0	58.0	0.782

### hnRNPD overexpression correlates with 14-3-3ζ and hnRNPK expression in OSCCs tissues

As shown above, both hnRNPK and 14-3-3ζ were identified in as interaction partners of hnRNPD in oral cancer cells and tissues. On the basis of IHC analysis, the immunoreactivity score ≥4 for both hnRNPD and hnRNPK, and a score ≥5 for 14-3-3**ζ** were considered as overexpression and were used in Kappa analysis. In order to confirm such association among clinical specimens of OSCCs, we performed Kappa analysis to determine the agreement of association between the hnRNPD, hnRNPK and 14-3-3ζ expressions in OSCCs using their IHC scores. One of the most important features of the Kappa statistics is that it is a measurement of the degree of agreement or reliability of agreement. Among OSCCs, 61 % agreement with a Kappa score (κ = 0.236) was observed between nuclear hnRNPD and hnRNPK (p = 0.0003) (Additional file 3: Table S3). Similar agreement with a Kappa score (κ = 0.248) was also observed between nuclear hnRNPD and 14-3-3ζ. This further strengthened our results of IP-LC–MS/MS. However, no significant agreement was observed between cytoplasmic expression of hnRNPD with hnRNPK or 14-3-3ζ in OSCCs.

## Discussion

The ability of proteins to form complexes by physically binding to each other and alterations in sub-cellular distribution lead to perturbations in the cell circuitry underlying cancer development. In this study, we determined hnRNPD associated protein networks to get an insight in molecular pathogenesis of oral cancer. Our network analysis revealed involvement of hnRNPD in multiple cellular pathways involved in progression and metastasis of oral cancer. We identified novel binding partners of hnRNPD suggesting its involvement in DNA repair, chromatin remodeling, RNAi mediated gene silencing and several other cell signaling pathways involved in cellular proliferation and apoptosis, besides its role in RNA processing and turnover. Several reports have demonstrated involvement of different RNA-binding proteins (RBPs) in determining the cellular fate of mRNA transcripts in terms of stability and rate of translation [11, 22]. In this respect, we observed other members of the hnRNP family including hnRNPA2/B1, hnRNPK, hnRNPU, hnRNPG forming heterodimers with hnRNPD in oral cancer cells. In this support, interaction of hnRNPK, hnRNPC, hnRNPL and hnRNPA2/B1 with hnRNPD in cervical and lung cancer cells has been reported earlier [31–33]. In addition, our results also suggested interaction of hnRNPD with other RNA binding proteins (RBPs) such as ELAVL1, RALY, EWSR1 and FUS in oral cancer. The fate of RNAs regulated by these protein interactions among hnRNPD and other RNA binding proteins in oral cancer is currently under investigation. However, a recent study reported RNA—dependent direct physical interaction between ELAVL1 and hnRNPD in the nucleus influences the expression of cyclin D1 and p16, both of which are important for oral cancer development [34, 35]. Besides RBPs, microRNAs are also important contributors to the post-transcriptional control of gene expression [36]. miRNAs act preferentially by binding to 3′-UTR region of target mRNA and are also involved in ARE-mediated mRNA instability. Precursor miRNAs are processed to mature miRNAs by multiprotein complexes including Drosha and Dicer and then incorporated into RNA induced silencing complex (RISC) [37]. We identified RNA polymerase II, eIF4 and argonaute proteins (EIF2C1, EIF2C2 and EIF2C3) that are important components of RISC in our interactome analysis of hnRNPD in oral cancer. hnRNPD associates with endogenous DICER1 mRNA and destabilizes it; however knockdown of hnRNPD using siRNA increased half-life of DICER1 mRNA and elevated its expression, while overexpression of hnRNPD lowered DICER1 mRNA and protein levels [38].

Interestingly, we also identified an important nuclear-cytoplasmic shuttling protein, 14-3-3ζ, a member of 14-3-3 family of proteins, as a binding partner of hnRNPD and verified their interaction in oral cancer cells (SCC4/MDA1986). Moreover, both hnRNPD and 14-3-3ζ co-localization was observed in cytoplasm of OSCC cells, suggesting 14-3-3ζ retains cytoplasmic hnRNPD, similar to 14-3-3σ. 14-3-3 family of proteins is known for their overlapping functions in orchestrating their target proteins in cytoplasm. Our results also showed presence of 14-3-3 binding motif in hnRNPD polypeptide and verified the interaction of 14-3-3ζ with hnRNPD may be dependent on Ser83 phosphorylation (present in this motif), unlike its interaction with 14-3-3σ, as reported earlier [39, 40]. Previous reports have shown binding of 14-3-3σ to hnRNPD masks its nuclear localization signal, retaining it in cytoplasm and enhances the rapid turnover of its target protein expression [39, 40]. Further, hnRNPD demonstrated significant association with 14-3-3ζ expression in clinical specimens of OSCCs analyzed in this study. In thyroid cancer, cytoplasmic hnRNPD interacts with mRNAs encoding cyclins (A1, B1, D1 and E1) and cyclin-dependent kinase inhibitors [41]. Stimulation of melanoma cells and monocytes by lipopolysaccharide resulted in cytoplasmic translocation of hnRNPD from nucleus and reduced levels of IL-6, IL-10 and TNF-α following activation of MKP-1 (MAPK phosphatase-1) [42–47]. This scenario might explain how nuclear-cytoplasmic translocation of hnRNPD can influence the cytoplasmic fate of mRNA.

Together, our network analysis suggested an important role of hnRNPD overexpression and its associated networks including protein interactions (direct/indirect) and regulation among these pathways is likely to play important role in determining the clinical outcome of OSCC patients. Supporting this hypothesis, our clinical findings demonstrated increased nuclear hnRNPD expression in clinical specimens of oral dysplasia and OSCCs as compared to normal oral mucosa. Notably, OSCC patients showing increased expression of nuclear hnRNPD had significantly reduced recurrence free survival. Nuclear hnRNPD overexpression in OSCCs and its emergence as a predictor of recurrence free survival in multivariate analysis in comparison with clinical and pathological parameters is an important novel finding in oral cancer, even though previous studies reported its overexpression in other malignancies including thyroid, melanoma, breast, cervix and murine lung tumors [4, 7, 16, 18, 48, 49, 35]. hnRNPD nuclear expression emerged as a significant predictor for recurrence with median RFS of 22 months as compared to patients who were node negative and had lower nuclear hnRNPD (median RFS = 69.0 months, p = 0.028). However, nuclear hnRNPD did not show any significant association with recurrence among OSCC patients with positive nodes at time of surgery. Thus, hnRNPD expression is likely to have the potential to define a high-risk subgroup among OSCC patients with nodal negative disease and might address the urgent need for more effective risk stratification strategies to improve patient care for these patients.

## Conclusions

Our interactome analysis of hnRNPD protein provided an insight into its novel functions in oral cancer cells. Further, nuclear hnRNPD emerged as a prognostic marker for evaluating the risk of recurrence in OSCC patients. Based on these results, we suggest nuclear hnRNPD as a potential target for molecular therapeutics for oral cancer in future.

## Additional files

Additional file 1:
**Figure S1.** (A) SDS-PAGE analysis of hnRNPD immunoprecipitates from OSCC samples. Lanes shows separation of IP-hnRNPD from oral cancer cells SCC4 and MDA1986, and beads only; PMW is protein molecular weight marker. (B) Binding partners of hnRNPD classified on basis of cellular function. Pie-chart demonstrating the distribution of binding partners of hnRNPD identified using IP-LC–MS/MS on the basis of their cellular functions. Additional file 2:
**Figure S2.** Spearman rank correlation co-efficient test:Graphs represent a positive correlation between (A) hnRNPD expression level and OSCC tumor size, (B) OSCC tumor stage. Additional file 3:
**Table S1.** List of key proteins identified as binding partners of hnRNPD in OSCC cells (SCC-4 / MDA1986) using IP-LC-MS/MS.
