# Supplementary figures and images for: Nuclear heterogeneous nuclear ribonucleoprotein D is associated with poor prognosis and interactome analysis reveals its novel binding partners in oral cancer
Source: J Transl Med. 2015 Aug 30;13:285. doi: 10.1186/s12967-015-0637-3 (PMC4553214; doi:10.1186/s12967-015-0637-3)

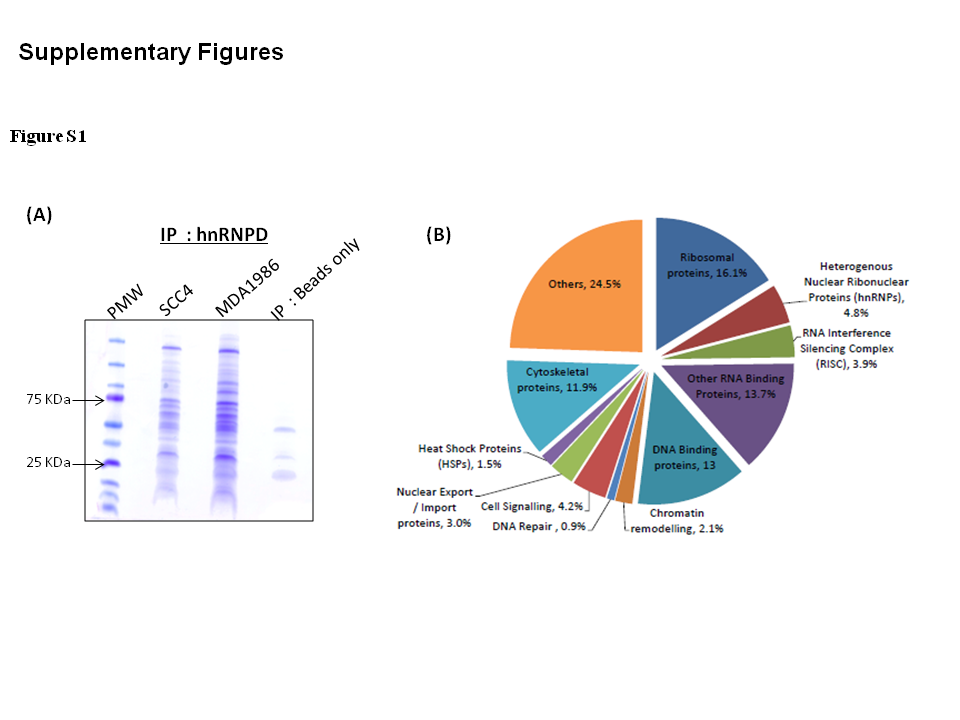

Supplement: Additional file 1: — Figure S1. (A) SDS-PAGE analysis of hnRNPD immunoprecipitates from OSCC samples. Lanes shows separation of IP-hnRNPD from oral cancer cells SCC4 and MDA1986, and beads only; PMW is protein molecular weight marker. (B) Binding partners of hnRNPD classified on basis of cellular function. Pie-chart demonstrating the distribution of binding partners of hnRNPD identified using IP-LC–MS/MS on the basis of their cellular functions. [file 12967_2015_637_MOESM1_ESM.tiff]

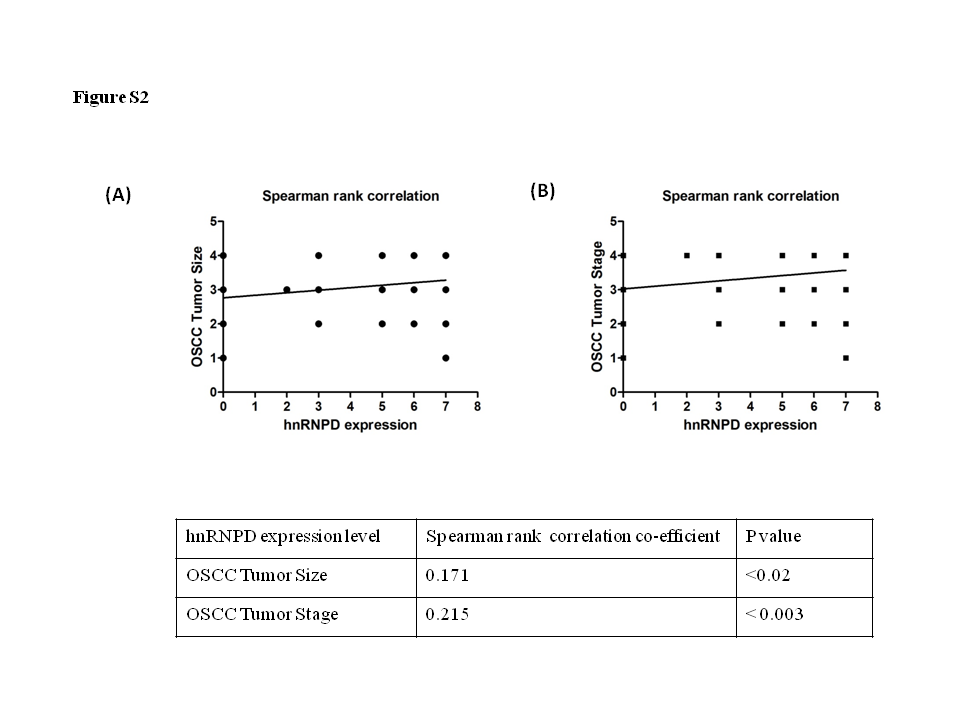

Supplement: Additional file 2: — Figure S2. Spearman rank correlation co-efficient test:Graphs represent a positive correlation between (A) hnRNPD expression level and OSCC tumor size, (B) OSCC tumor stage. [file 12967_2015_637_MOESM2_ESM.tiff]
