# Supplementary material for: Nuclear heterogeneous nuclear ribonucleoprotein D is associated with poor prognosis and interactome analysis reveals its novel binding partners in oral cancer
Source: J Transl Med. 2015 Aug 30;13:285. doi: 10.1186/s12967-015-0637-3 (PMC4553214; doi:10.1186/s12967-015-0637-3)
Supplement: Additional file 3: — Table S1. List of key proteins identified as binding partners of hnRNPD in OSCC cells (SCC-4 / MDA1986) using IP-LC-MS/MS. [file 12967_2015_637_MOESM3_ESM.doc]

**Table S1.** List of key proteins identified as binding partners of hnRNPD in OSCC cells (SCC-4 / MDA1986) using IP-LC-MS/MS

| **UniProtKB/Swiss-Prot ID** | 1. **RNA Binding proteins (RBPs)** |  |
| --- | --- | --- |
|  | **I ) Heterogenous Nuclear Ribonuclear Proteins (hnRNPs)** |  |
| Q99729 | Heterogeneous nuclear ribonucleoprotein A/B | HNRNPAB |
| Q13151 | Heterogeneous nuclear ribonucleoprotein A0 | HNRNPA0 |
| P09651 | Heterogeneous nuclear ribonucleoprotein A1 | HNRNPA1 |
| P51991 | Heterogeneous nuclear ribonucleoprotein A3 | HNRNPA3 |
| **Q14103** | **Heterogeneous nuclear ribonucleoprotein D0** | **HNRNPD** |
| P52597 | Heterogeneous nuclear ribonucleoprotein F | HNRNPF |
| P38159 | Heterogeneous nuclear ribonucleoprotein G | RBMX |
| P31943 | Heterogeneous nuclear ribonucleoprotein H | HNRNPH1 |
| P31942 | Heterogeneous nuclear ribonucleoprotein H3 | HNRNPH3 |
| P61978 | Heterogeneous nuclear ribonucleoprotein K | HNRNPK |
| P14866 | Heterogeneous nuclear ribonucleoprotein L | HNRNPL |
| P52272 | Heterogeneous nuclear ribonucleoprotein M | HNRNPM |
| O60506 | Heterogeneous nuclear ribonucleoprotein Q | SYNCRIP |
| O43390 | Heterogeneous nuclear ribonucleoprotein R | HNRNPR |
| Q00839 | Heterogeneous nuclear ribonucleoprotein U | HNRNPU |
| P22626 | Heterogeneous nuclear ribonucleoproteins A2/B1 | HNRNPA2B1 |
| P07910 | Heterogeneous nuclear ribonucleoproteins C1/C2 | HNRNPC |
|  |  |  |
|  | **II) RNA Induced Silencing Complex (RISC)** |  |
| O60573 | Eukaryotic translation initiation factor 4E type 2 | EIF4E2 |
| P24928 | DNA-directed RNA polymerase II subunit RPB1 | POLR2A |
| P30876 | DNA-directed RNA polymerase II subunit RPB2 | POLR2B |
| Q93074 | Mediator of RNA polymerase II transcription subunit 12 | MED12 |
| O60244 | Mediator of RNA polymerase II transcription subunit 14 | MED14 |
| Q96RN5 | Mediator of RNA polymerase II transcription subunit 15 | MED15 |
| Q9Y2X0 | Mediator of RNA polymerase II transcription subunit 16 | MED16 |
| Q9NVC6 | Mediator of RNA polymerase II transcription subunit 17 | MED17 |
| O75448 | Mediator of RNA polymerase II transcription subunit 24 | MED24 |
| Q9NPJ6 | Mediator of RNA polymerase II transcription subunit 4 | MED4 |
| Q9UL18 | Protein argonaute-1 | EIF2C1 |
| Q9UKV8 | Protein argonaute-2 | EIF2C2 |
| Q9H9G7 | Protein argonaute-3 | EIF2C3 |
|  |  |  |
|  | **III) Other RBPs** |  |
| Q15717 | ELAV-like protein 1 | ELAVL1 |
| Q6PKG0 | La-related protein 1 | LARP1 |
| Q01844 | RNA-binding protein EWS | EWSR1 |
| P35637 | RNA-binding protein FUS | FUS |
| Q9UKM9 | RNA-binding protein Raly | RALY |
| P19338 | Nucleolin | NCL |
|  |  |  |
|  | 1. **DNA Binding proteins** |  |
|  | 1. **Histones** |  |
| Q02539 | Histone H1.1 | HIST1H1A |
| P16403 | Histone H1.2 | HIST1H1C |
| P16402 | Histone H1.3 | HIST1H1D |
| P10412 | **Histone H1.4** | HIST1H1E |
| P16401 | Histone H1.5 | HIST1H1B |
| Q92522 | Histone H1x | H1FX |
| P04908 | Histone H2A type 1 | HIST1H2AG |
| Q96QV6 | Histone H2A type 1-A | HIST1H2AA |
| P04908 | Histone H2A type 1-B/E | HIST1H2AB |
| Q93077 | Histone H2A type 1-C | HIST1H2AC |
| P20671 | Histone H2A type 1-D | HIST1H2AD |
| Q96KK5 | Histone H2A type 1-H | HIST1H2AH |
| Q99878 | Histone H2A type 1-J | HIST1H2AJ |
| Q6FI13 | Histone H2A type 2-A | HIST2H2AA3 |
| Q8IUE6 | Histone H2A type 2-B | HIST2H2AB |
| Q16777 | Histone H2A type 2-C | HIST2H2AC |
| Q7L7L0 | Histone H2A type 3 | HIST3H2A |
| Q9BTM1 | Histone H2A.J | H2AFJ |
| P16104 | Histone H2A.x | H2AFX |
| P62807 | Histone H2B type 1-C/E/F/G/I | HIST1H2BC |
| P58876 | Histone H2B type 1-D | HIST1H2BD |
| Q93079 | Histone H2B type 1-H | HIST1H2BH |
| O60814 | Histone H2B type 1-K | HIST1H2BK |
| Q99880 | Histone H2B type 1-L | HIST1H2BL |
| Q99877 | Histone H2B type 1-M | HIST1H2BM |
| Q99876 | Histone H2B type 1-N | HIST1H2BN |
| P57053 | Histone H2B type 2-F | HIST2H2BF |
| P57053 | Histone H2B type F-S | H2BFS |
| P62805 | Histone H4 | HIST1H4A |
| Q86X55 | Histone-arginine methyltransferase CARM1 | CARM1 |
| Q7Z2W4 | Zinc finger CCCH-type antiviral protein 1 | ZC3HAV1 |
| Q6NZY4 | Zinc finger CCHC domain-containing protein 8 | ZCCHC8 |
| Q9ULJ6 | Zinc finger MIZ domain-containing protein 1 | ZMIZ1 |
| O43670 | Zinc finger protein 207 | ZNF207 |
| Q92785 | Zinc finger protein ubi-d4 | DPF2 |
|  |  |  |
|  | 1. **Chromatin Remodelling** |  |
| Q92922 | SWI/SNF complex subunit SMARCC1 | SMARCC1 |
| Q8TAQ2 | SWI/SNF complex subunit SMARCC2 | SMARCC2 |
| Q12824 | SWI/SNF-related matrix-associated actin-dependent regulator of chromatin subfamily B member 1 | SMARCB1 |
| Q92925 | SWI/SNF-related matrix-associated actin-dependent regulator of chromatin subfamily D member 1 | SMARCD1 |
| Q92925 | SWI/SNF-related matrix-associated actin-dependent regulator of chromatin subfamily D member 2 | SMARCD2 |
| Q969G3 | SWI/SNF-related matrix-associated actin-dependent regulator of chromatin subfamily E member 1 | SMARCE1 |
|  |  |  |
|  | 1. **DNA Repair** |  |
| P13010 | X-ray repair cross-complementing protein 5 | XRCC5 |
| P12956 | X-ray repair cross-complementing protein 6 | XRCC6 |
| P09874 | Poly [ADP-ribose] polymerase 1 | PARP1 |
|  |  |  |
|  | **(C) Cell Signalling proteins** |  |
| P27348 | 14-3-3 protein zeta / theta | YWHAZ / YWHAQ |
| P49336 | Cell division protein kinase 8 | CDK8 |
| P24863 | Cyclin-C | CCNC |
| Q9H3D4 | Tumor protein 63 | TP63 |
| Q13283 | Ras GTPase-activating protein-binding protein 1 | G3BP1 |
| Q9UN86 | Isoform B of Ras GTPase-activating protein-binding protein 2 | G3BP2 |
| P63244 | Guanine nucleotide-binding protein subunit beta-2-like 1 | GNB2L1 |
| P62136 | Serine/threonine-protein phosphatase PP1-alpha catalytic subunit | PPP1CA |
| P62140 | Serine/threonine-protein phosphatase PP1-beta catalytic subunit | PPP1CB |
| P06702 | Protein S100-A9 | S100A9 |

**Table S2. Canonical pathways revealed by Ingenuity pathway analysis of proteins identified in the hnRNPD interactome**

| **Canonical Pathways** |  |  |  |
| --- | --- | --- | --- |
| **Ingenuity Canonical Pathways** | **-log(p-value)** | **Ratio** | **Molecules** |
| EIF2 Signaling | 12.7 | 0.0896 | RPL24,RPL22,RPL4,RPS19,PPP1CB,RPL23,RPL9, RPS7,RPL15,AGO3,RPL10,RPL19,RPLP2,PPP1CA,AGO1,RPL29,RPS24,RPSA |
| Cleavage and Polyadenylation of Pre-mRNA | 11.3 | 0.538 | CPSF2,NUDT21,PABPN1,CPSF3,CSTF3,CPSF4, WDR33 |
| Estrogen Receptor Signaling | 8.46 | 0.0882 | POLR2A,CDK8,CCNC,MED17,MED15,MED16, POLR2B,MED12,MED24,MED4,MED14,NCOA3 |
| Tight Junction Signaling | 3.15 | 0.0419 | CPSF2,NUDT21,MYH14,YBX3,CPSF3,CSTF3, CPSF4 |
| Virus Entry via Endocytic Pathways | 2.78 | 0.0495 | FLNB,AP2B1,AP2A1,FLNA,CLTC |
| Regulation of eIF4 and p70S6K Signaling | 2.51 | 0.0343 | RPS7,AGO3,RPS19,AGO1,RPSA,RPS24 |
| Clathrin-mediated Endocytosis Signaling | 2.04 | 0.0303 | MYO6,AP2B1,AP2A1,SNX9,CLTC,CTTN |
| Granzyme A Signaling | 1.85 | 0.1 | H1FX,HIST1H1A |
| Huntington's Disease Signaling | 1.68 | 0.0238 | POLR2A,GNB2L1,CLTC,POLR2B,HSPA5,HSPA2 |
| Lipid Antigen Presentation by CD1 | 1.59 | 0.0667 | AP2B1,AP2A1 |
| Retinoic acid Mediated Apoptosis Signaling | 1.57 | 0.0411 | ZC3HAV1,DAP3,PARP1 |
| Protein Kinase A Signaling | 1.51 | 0.0197 | YWHAQ,FLNB,FLNA,H1FX,GNB2L1,HIST1H1A, PPP1CB,PPP1CA |
| Proline Biosynthesis I | 1.42 | 0.0714 | PYCRL |
| Glucocorticoid Receptor Signaling | 1.39 | 0.0201 | POLR2A,POLR2B,HSPA5,HSPA2,MED14,NCOA3 |
| Nucleotide Excision Repair Pathway | 1.35 | 0.0556 | POLR2A,POLR2B |
| Proline Biosynthesis II (from Arginine) | 1.33 | 0.05 | PYCRL |
| CTLA4 Signaling in Cytotoxic T Lymphocytes | 1.28 | 0.0312 | AP2B1,AP2A1,CLTC |
| Arginine Degradation VI (Arginase 2 Pathway) | 1.25 | 0.0625 | PYCRL |
| Systemic Lupus Erythematosus Signaling | 1.19 | 0.0195 | SNRPN,SNRPB,SNRPD1,HNRNPC,SNRPA1 |
| Role of Oct4 in Mammalian Embryonic Stem Cell Pluripotency | 1.16 | 0.0385 | IGF2BP1,PARP1 |
| GABA Receptor Signaling | 1.12 | 0.0357 | AP2B1,AP2A1 |
| Assembly of RNA Polymerase II Complex | 1.08 | 0.0357 | POLR2A,POLR2B |
| Androgen Signaling | 1.03 | 0.0207 | POLR2A,GNB2L1,POLR2B |
| Hereditary Breast Cancer Signaling | 0.995 | 0.0224 | POLR2A,H2AFX,POLR2B |
| Purine Nucleotides De Novo Biosynthesis II | 0.961 | 0.0238 | IMPDH2 |
| mTOR Signaling | 0.948 | 0.0188 | RPS7,RPS19,RPSA,RPS24 |
| Breast Cancer Regulation by Stathmin1 | 0.936 | 0.0187 | TUBB4B,GNB2L1,PPP1CB,PPP1CA |
| Urate Biosynthesis/Inosine 5'-phosphate Degradation | 0.928 | 0.0455 | IMPDH2 |
| DNA Double-Strand Break Repair by Non-Homologous End Joining | 0.898 | 0.05 | PARP1 |
| Methionine Degradation I (to Homocysteine) | 0.87 | 0.0435 | PRMT1 |
| Remodeling of Epithelial Adherens Junctions | 0.857 | 0.0286 | TUBB4B,CTNND1 |
| Cardiac Î²-adrenergic Signaling | 0.851 | 0.019 | GNB2L1,PPP1CB,PPP1CA |
| Granzyme B Signaling | 0.844 | 0.0556 | PARP1 |
| Cysteine Biosynthesis III (mammalia) | 0.82 | 0.0333 | PRMT1 |
| Caveolar-mediated Endocytosis Signaling | 0.798 | 0.0235 | FLNB,FLNA |
| Purine Nucleotides Degradation II (Aerobic) | 0.797 | 0.0286 | IMPDH2 |
| Endoplasmic Reticulum Stress Pathway | 0.797 | 0.0526 | HSPA5 |
| Dopamine Receptor Signaling | 0.78 | 0.0208 | PPP1CB,PPP1CA |
| Epithelial Adherens Junction Signaling | 0.78 | 0.0195 | TUBB4B,MYH14,CTNND1 |
| VDR/RXR Activation | 0.763 | 0.0227 | CCNC,NCOA3 |
| Aldosterone Signaling in Epithelial Cells | 0.744 | 0.0179 | HSPD1,HSPA5,HSPA2 |
| Salvage Pathways of Pyrimidine Ribonucleotides | 0.706 | 0.0194 | PYCRL,CDK8 |
| CDK5 Signaling | 0.683 | 0.0206 | PPP1CB,PPP1CA |
| Apoptosis Signaling | 0.683 | 0.02 | LMNA,PARP1 |
| UVA-Induced MAPK Signaling | 0.662 | 0.0204 | ZC3HAV1,PARP1 |
| Cell Cycle Control of Chromosomal Replication | 0.625 | 0.0294 | MCM5 |
| CREB Signaling in Neurons | 0.625 | 0.0145 | POLR2A,GNB2L1,POLR2B |
| PPARÎ±/RXRÎ± Activation | 0.62 | 0.0151 | MED12,MED24,NCOA3 |
| Superpathway of Methionine Degradation | 0.599 | 0.0156 | PRMT1 |
| ILK Signaling | 0.571 | 0.0146 | FLNB,FLNA,MYH14 |
| ERK/MAPK Signaling | 0.567 | 0.0142 | YWHAQ,PPP1CB,PPP1CA |
| G Protein Signaling Mediated by Tubby | 0.553 | 0.0227 | GNB2L1 |
| Interferon Signaling | 0.553 | 0.0278 | MED14 |
| Cell Cycle Regulation by BTG Family Proteins | 0.542 | 0.0256 | PRMT1 |
| CCR3 Signaling in Eosinophils | 0.518 | 0.0149 | GNB2L1,PPP1CB |
| 14-3-3-mediated Signaling | 0.508 | 0.0165 | YWHAQ,TUBB4B |
| Synaptic Long Term Potentiation | 0.493 | 0.0154 | PPP1CB,PPP1CA |
| Actin Cytoskeleton Signaling | 0.47 | 0.0124 | FLNA,MYH14,PPP1CB |
| Cell Cycle: G2/M DNA Damage Checkpoint Regulation | 0.46 | 0.0204 | YWHAQ |
| Cellular Effects of Sildenafil (Viagra) | 0.453 | 0.0129 | MYH14,PPP1CB |
| eNOS Signaling | 0.444 | 0.0129 | HSPA5,HSPA2 |
| Insulin Receptor Signaling | 0.44 | 0.0134 | PPP1CB,PPP1CA |
| Myc Mediated Apoptosis Signaling | 0.367 | 0.0159 | YWHAQ |
| Induction of Apoptosis by HIV1 | 0.361 | 0.0149 | SLC25A5 |
| ATM Signaling | 0.356 | 0.0152 | H2AFX |
| Germ Cell-Sertoli Cell Junction Signaling | 0.353 | 0.0118 | TUBB4B,CTNND1 |
| Protein Ubiquitination Pathway | 0.351 | 0.0111 | HSPD1,HSPA5,HSPA2 |
| GM-CSF Signaling | 0.341 | 0.0147 | GNB2L1 |
| Antiproliferative Role of Somatostatin Receptor 2 | 0.336 | 0.0139 | GNB2L1 |
| ERK5 Signaling | 0.336 | 0.0147 | YWHAQ |
| Dopamine-DARPP32 Feedback in cAMP Signaling | 0.331 | 0.0107 | PPP1CB,PPP1CA |
| Pyridoxal 5'-phosphate Salvage Pathway | 0.331 | 0.0133 | CDK8 |
| Agrin Interactions at Neuromuscular Junction | 0.317 | 0.0143 | CTTN |
| Chemokine Signaling | 0.312 | 0.0133 | PPP1CB |
| CCR5 Signaling in Macrophages | 0.308 | 0.0103 | GNB2L1 |
| RAR Activation | 0.301 | 0.0105 | PARP1,PRMT1 |
| Sertoli Cell-Sertoli Cell Junction Signaling | 0.293 | 0.0101 | TUBB4B,YBX3 |
| Ephrin B Signaling | 0.291 | 0.0122 | GNB2L1 |
| Production of Nitric Oxide and Reactive Oxygen Species in Macrophages | 0.288 | 0.00943 | PPP1CB,PPP1CA |
| Calcium Signaling | 0.285 | 0.00922 | TP63,MYH14 |
| Role of Wnt/GSK-3Î² Signaling in the Pathogenesis of Influenza | 0.272 | 0.012 | NCOA3 |
| Regulation of Actin-based Motility by Rho | 0.261 | 0.011 | PPP1CB |
| Thrombin Signaling | 0.259 | 0.00948 | GNB2L1,PPP1CB |
| TR/RXR Activation | 0.251 | 0.00917 | NCOA3 |
| Integrin Signaling | 0.246 | 0.00962 | PPP1CB,CTTN |
| Leukocyte Extravasation Signaling | 0.244 | 0.00952 | CTTN,CTNND1 |
| Î±-Adrenergic Signaling | 0.241 | 0.00917 | GNB2L1 |
| G Beta Gamma Signaling | 0.238 | 0.00826 | GNB2L1 |
| p53 Signaling | 0.226 | 0.00926 | TP63 |
| IL-1 Signaling | 0.226 | 0.00917 | GNB2L1 |
| IGF-1 Signaling | 0.215 | 0.00935 | YWHAQ |

**Table S3:** Kappa Analysis revealed the agreement of association between hnRNPD and hnRNPK or 14-3-3ζ.

Overall Association

| **hnRNPD Cytoplasmic** | **hnRNPK Cyto** | **14-3-3ᵹ Cyto** |  |
| --- | --- | --- | --- |
|  | 0.9696 | 0.9296 | P value |
|  | -0.1189 (65.41%) | -0.0607 (42.11%) | Kappa (% agreement) |
| **hnRNPD Nuclear** | **hnRNPK Nuc** | **14-3-3 ᵹ Nuc** |  |
|  | 0.0003 | 0.0002 | P value |
|  | 0.2362 (61.22%) | 0.2476 (62.24%) | Kappa (% agreement) |
